# Analysis of the risk of death and its associated risk factors in Chinese patients with young-onset type 2 diabetes

**DOI:** 10.3389/fendo.2024.1451364

**Published:** 2024-12-19

**Authors:** Dan Wu, Yang Yu, Haoran Zheng, Ling Xiang, Xiaoqing Wang, Yong Zhang, Zhongming Sun, Dandan Miao, Jinyi Zhou, Enchun Pan, Wen Hu

**Affiliations:** ^1^ Department of Endocrinology, Huai’an Hospital Affiliated to Xuzhou Medical University and Huai’an Second People’s Hospital, Huai’an, Jiangsu, China; ^2^ Department of Chronic Disease Prevention and Control, Huai’an City Center for Disease Control and Prevention, Huai’an, China; ^3^ Department of Non-communicable Chronic Disease Control, Jiangsu Provincial Center for Disease Control and Prevention, Nanjing, China

**Keywords:** young-onset type 2 diabetes, Chinese patients, all-cause mortality, risk factors, sleep duration

## Abstract

**Objectives:**

To examine the association between the age at onset of diabetes and the risk of all-cause mortality in a population of individuals diagnosed with type 2 diabetes mellitus (T2DM) and to identify risk factors associated with all-cause mortality in young-onset T2DM (YOD) patients in China.

**Methods:**

This study utilized a cohort of 9759 patients who were diagnosed with T2DM and who were registered and enrolled in the National Basic Public Health Service Management Program in Qinghe District (now Qingjiangpu District) and Huai’an District, Huai’an City, Jiangsu Province, China. The patients were observed from November 2013 to July 2014, and all-cause mortality data were obtained by comprehensive matching with the Huai’an City Resident Mortality Database as of December 31, 2019. A Cox proportional hazards model was used to compute the hazard ratios (HRs) and their corresponding 95% confidence intervals (95% CIs) for all-cause mortality risk during the follow-up period among patients with varying disease onset ages. Additionally, subgroup analyses were conducted based on sex, age, lifestyle factors, and baseline clinical parameters.

**Results:**

A total of 7572 patients with T2DM, including 2874 men and 4698 women aged 57.9 ± 8.0 years, were ultimately included in the study. 1) At baseline, a greater proportion of YOD patients were engaged in high-intensity activities, had a longer sleep duration, had a longer duration of T2DM, had a family history of T2DM, had microvascular complications (kidney disease, retinopathy, neuropathy, diabetic foot, etc.), and received glucose-lowering treatment. Moreover, patients in the YOD group also had significantly greater baseline HbA1c, FBG, and estimated glomerular filtration rate (eGFR) than did those in the onset at 41-60 years (MD) group and the onset at 61-75 years (SD) group. 2) During the six-year follow-up period, a total of 1057 deaths were documented. After adjusting for confounding factors and utilizing SD as the reference group, the HRs for deaths occurring in the YOD and MD groups were 1.383 (95% CI: 0.717-2.667) and 1.006 (95% CI: 0.763-1.326), respectively. Moreover, the risk of death in the YOD group remained highest in the sensitivity analysis that excluded patients with coronary heart disease at baseline, stroke patients, and patients who died within the first three years of follow-up. 3) Sleep duration was identified as an independent risk factor for mortality in the YOD group, with a notable increase in the risk of all-cause mortality when sleep duration exceeded 9 hours per day.

**Conclusion:**

The risk of all-cause mortality in YOD patients was 1.38 times greater than that in MD and SD patients, and the longer the sleep duration was, the greater the risk of death, especially when sleep duration exceeded 9 hours per day.

## Introduction

In China, the number of diabetes patients ranks first in the world, reaching 141 million people, and the incidence rate is as high as 12.8% (1). Notably, the incidence of type 2 diabetes mellitus (T2DM) among younger people has experienced a notable surge in recent years. In the United States, the prevalence of youth-onset T2DM has increased annually by 4.8%, increasing from 9.0 per 100,000 individuals in 2003 to 13.8 per 100,000 individuals in the years 2014-2015 (2). In Canada, there was a notable increase in the incidence of T2DM among children, with rates increasing from 0.17 to 0.57 per 100 individuals between 1995 and 2015 (3). A comprehensive epidemiological investigation conducted in 2013, encompassing a sample size of 179,347 individuals, revealed that the prevalence of T2DM among individuals aged 18 to 40 in China has increased to 5.9%. This alarming trend has imposed a significant health burden on the younger population, necessitating urgent attention (4).

The occurrence of T2DM at an early stage leads to an extended period of hyperglycemia exposure, consequently increasing vulnerability to long-term complications. According to the TODAY study, the cumulative incidence of microvascular complications reaches 50.0% after 9 years and can increase to 80.1% after 15 years (5). A comprehensive analysis of 26 observational cohorts involving a total of 1,325,493 individuals from 30 countries globally revealed that each additional year in the age at which T2DM is diagnosed is associated with a 4% decrease in the risk of all-cause mortality, a 3% decrease in the risk of macrovascular disease, and a 5% decrease in the risk of microvascular disease (6). Research has demonstrated that early-onset T2DM (YOD, diagnosed prior to the age of 40 years) generally manifests as a more severe and aggressive disease phenotype (7). This finding implies that the progression of T2DM in young adults who develop the disease at an early age may be more accelerated and severe than that in young adults who develop it later in life. Additionally, it may result in premature mortality and therefore necessitates clinical intervention (8, 9). However, there are few studies on the correlation between T2DM and mortality in young adults upon disease onset, particularly within the Chinese population. Therefore, it is imperative to conduct further investigations into the risk factors associated with mortality in this specific group of young adults with T2DM to mitigate the significant increase in mortality rates.

This study utilized follow-up data from T2DM patients in Huai’an City to examine the correlation between the age at onset of T2DM and mortality risk and to identify risk factors associated with all-cause mortality in YOD patients. The objective was to enhance the clinical diagnosis and treatment of this population, thereby improving patients’ quality of life and reducing complications, ultimately mitigating the risk of mortality in this cohort.

## Methods

### Ethics statement

The study was reviewed by the Ethics Committee of the Jiangsu Provincial CDC (approval number: 2013026), and all respondents signed an informed consent form before the formal investigation.

### Study population

The inclusion criteria encompassed a total of 9759 patients diagnosed with T2DM who were duly registered and enrolled in the National Basic Public Health Service Management Program. This study specifically focused on patients residing in Qinghe District (now Qingjiangpu District) and Huai’an District, located in Huai’an City, Jiangsu Province, China, during the period spanning from November 2013 to July 2014.

**Figure 1:**
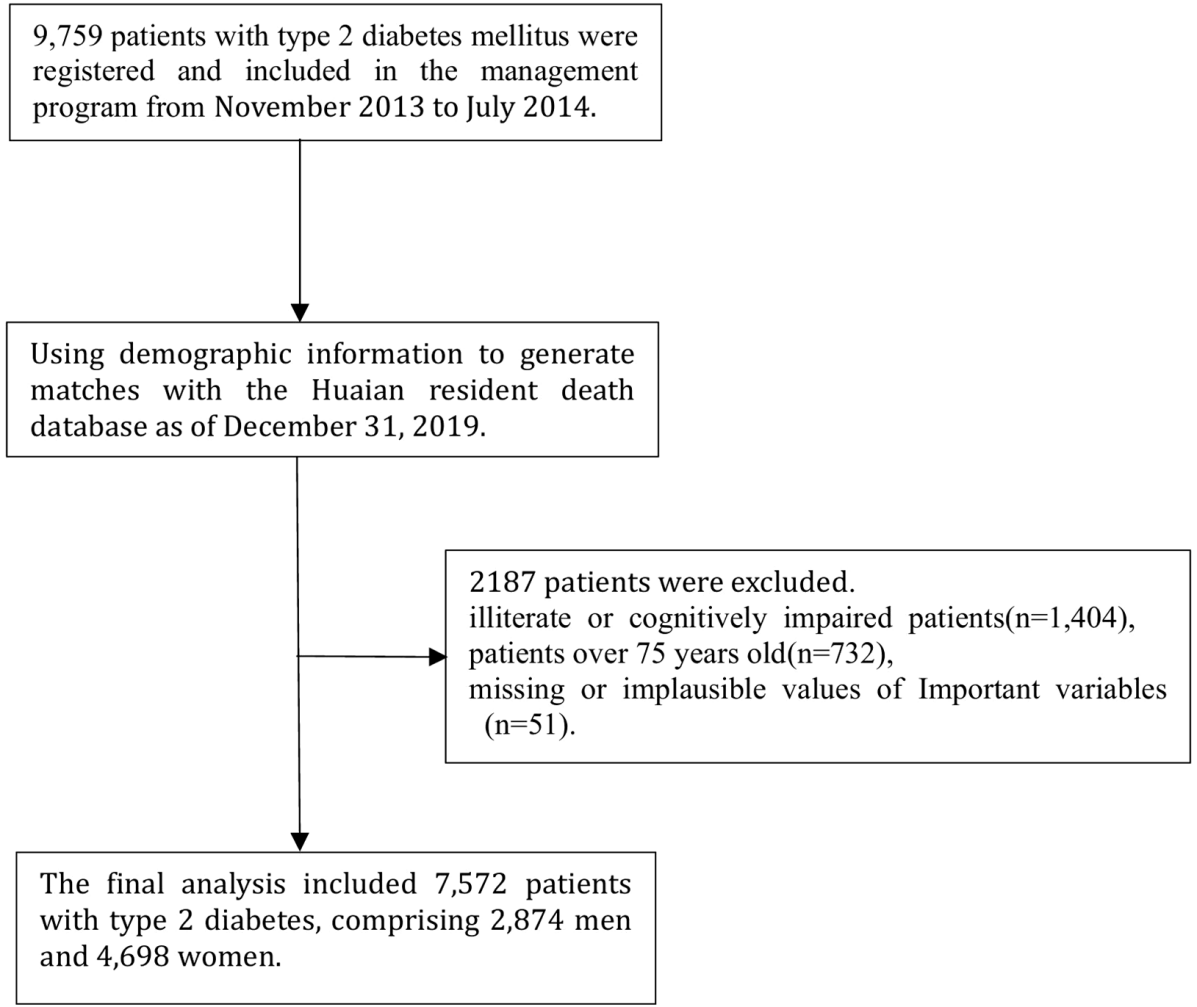
Selection process for participants.

A comprehensive exclusion process was conducted, resulting in the exclusion of a total of 2,187 subjects. Among these exclusions, 1,404 patients were excluded due to cognitive impairment or illiteracy, 732 patients were excluded due to an actual age exceeding 75 years, and 51 patients were excluded due to the absence or implausibility of values in key variables. As a result, the analysis included a total of 7,572 patients diagnosed with T2DM (**Figure 1**). The follow-up period extended until December 31, 2019, with the endpoints being death, loss to follow-up, or completion of the study.

### Data collection

#### Questionnaire survey

The questionnaires utilized in this study were standardized based on the individual questionnaire developed by the Jiangsu Provincial Center for Disease Control and Prevention for the Comprehensive Community Diabetes Intervention and Application Project. These questionnaires encompassed various aspects, such as general demographic and sociological information, health-related behaviors, and disease history. The data collection process involved conducting face-to-face interviews with personnel who had received consistent training and successfully passed an examination.

#### Physical measurement

The physical measurements primarily included height, weight, and blood pressure (BP). Height measurements were taken in a quiet, spacious area with a level surface. Participants removed shoes, hats, and outerwear, and those with long hair wore it down. The patients stood upright with their knees together and straight, their eyes forward, and their head held steady. The investigator lowered the head plate until it touched the top of the head and then read the measurement to the nearest 0.1 cm. The respondents were instructed to wear one layer of clothing and remove any belongings before standing in the center of the weighing plate with their feet positioned symmetrically. The participants were asked to stand upright with their arms hanging naturally and their head straight. The weight was recorded to the nearest 0.1 kg after the scale reading stabilized. Blood pressure was measured using a digital blood pressure monitor (Omron HBP-1300, Kyoto, Japan), with patients instructed to refrain from exercise or eating for one hour prior to measurement and to rest for five minutes before the readings were taken. The left elbow was positioned on the table, the palm was positioned up, the arm band was positioned above the elbow joint, and the air tube was placed in line with the middle finger. The armband was placed at heart level, the electronic sphygmomanometer was started, pressurization and depressurization were allowed, and the blood pressure was recorded in mmHg. Three measurements were taken with a 1-minute break between each measurement, and the average of the last two measurements was calculated.

#### Laboratory test

The laboratory testing procedures included on-site collection and processing of samples, preservation and transportation of samples, and testing of blood specimens. Fasting blood samples were obtained in the early morning, followed by centrifugation and division of the blood specimens. Subsequently, the uniformly divided specimens were transported to Nanjing Jinwei Medical Testing Center for comprehensive analysis of fasting blood glucose (FBG), glycated hemoglobin (HbA1c), blood lipids, liver function, renal function, and other relevant indices. The measurement of HbA1c was conducted using ion-exchange high-performance liquid chromatography (HPLC), which was certified by the National Glycohemoglobin Standardization Program (NGSP) and the International Federation of Clinical Chemistry and Laboratory Medicine (IFCC). The analysis was performed on a Bio-Rad D-10^®^ autoanalyzer (Bio-Rad Laboratories, CA, USA). FBG levels were measured using the hexokinase method with a Roche Cobas c701 automatic analyzer (Roche Diagnostics, Mannheim, Germany), while lipid levels were assessed using the enzyme method on the same analyzer.

#### Index definition

T2DM was defined as an FBG level of ≥7.0 mmol/L or a 2-hour postprandial blood glucose level of ≥11.1 mmol/L, accompanied by a self-reported history of T2DM and the exclusion of type 1 diabetes mellitus. All individuals diagnosed with T2DM in this study were identified at the township or community level, as well as at hospitals of higher tiers (10).

Hypertension was defined as having a systolic blood pressure of 140 mmHg or greater, a diastolic blood pressure of 90 mmHg or greater, or a diagnosis of hypertension from a district/county level hospital or higher. To ensure accuracy, blood pressure measurements were taken three times and averaged (11).

Stroke was diagnosed based on the technical and industry diagnostic criteria set forth by the Chinese Center for Cardiovascular Diseases or in a hospital at the district/county level or higher (12).

Renal disease referred to the presence of structural or functional abnormalities in the kidneys, characterized by a glomerular filtration rate (GFR) of less than 60 mL/min/1.73 m^2^ or proteinuria equal to or exceeding 30 mg/24 hours, persisting for a duration exceeding three months (13).

Coronary atherosclerotic heart disease, as indicated by coronary angiography, was characterized by the presence of at least 50% stenosis in the internal diameter of one or more major coronary arteries (including the left main stem, left anterior descending, left echogenic, and right coronary arteries) or their significant branches (such as the first diagonal, second diagonal, obtuse marginal, acute marginal, left ventricular posterior, and posterior descending branches) (14).

The duration of sleep was assessed by means of an in-person, on-site inquiry in which participants were asked, “On an average day, how many hours of sleep do you typically obtain?” Subsequently, the relevant data were collected. The term “smoking” was operationally defined as individuals who had smoked a minimum of 100 cigarettes from the initiation of their smoking behavior until the current point in time. Drinking was characterized as consuming alcohol at a frequency of once a month or more, on average, and persisting in this behavior at the time of the inquiry. High-intensity physical activity refers to tasks involving substantial physical exertion, such as lifting heavy objects or engaging in plowing activities, which necessitate a notable increase in respiration or heart rate and last for at least 10 minutes. The duration of T2DM was the length of time between the date of the baseline survey and the date of the first diagnosis of T2DM.

### Criteria for grouping

The patients were classified into three distinct groups based on the age at which they were initially diagnosed with T2DM. These groups included 1) the YOD group (n=718), young onset of T2DM diagnosed at or before the age of 40; 2) the MD group (n=5086), midlife T2DM diagnosed between the ages of 41 and 60; and 3) the SD group (n=1768), senior T2DM diagnosed between the ages of 61 and 75.

### Ascertainment of mortality

In this study, the demographic information of 9759 patients with T2DM included in the study was comprehensively matched with the death database of Huai ‘an residents on December 31, 2019, to obtain all-cause death information as the end point of this study.

### Statistical analysis

The statistical software IBM SPSS Statistics version 20 was used to analyze the data. The normality of the measurement data was assessed using the Kolmogorov−Smirnov test, while the median (quartiles) was utilized to represent the data that did not adhere to a normal distribution. Group comparisons were conducted using the Kruskal−Wallis H test, whereas the chi-square test was used to compare categorical count data. Additionally, the chi-square test was used to compare data between two groups. The present study employed the Cox proportional hazards model to examine the impact of age at onset on the risk of mortality in individuals diagnosed with T2DM. The hazard ratio (HR) values, along with their corresponding 95% confidence intervals (CIs), were calculated. All test levels were found to be statistically significant at a threshold of P<0.05. Additionally, stepwise adjustments were performed to account for potential confounding factors that are recognized or anticipated to influence the risk of death. To mitigate the potential influence of reverse causality stemming from preexisting cardiovascular and cerebrovascular disease, as well as other underlying conditions, sensitivity analyses were conducted. These analyses involved the exclusion of patients with T2DM who exhibited coronary heart disease and stroke at the outset, as well as those who passed away within the first three years of follow-up. The resulting disparity was deemed statistically significant at a significance level of P<0.05.

## Results

### Baseline characteristics of patients

A total of 7572 patients with T2DM, including 2874 men and 4698 women aged 57.9 ± 8.0 years, were ultimately included in the study. The variables of alcohol consumption, body mass index (BMI), waist-to-hip ratio (WHR), and triglyceride (TG) levels did not exhibit statistically significant differences across the various age groups at disease onset (p>0.05). However, all other parameters demonstrated significant differences (p<0.01).

A general data comparison revealed that the proportions of patients who engaged in high-intensity activities, had a long sleep duration, had a long duration of T2DM, had a family history of T2DM, had microvascular complications (kidney disease, retinopathy, neuropathy, diabetic foot, etc.), and who received glucose-lowering treatment were greater in the YOD group than in the MD and SD groups. However, there was a lower age at onset, proportion of females, smoking rate, and proportion of macrovascular complications (including hypertension, coronary heart disease, and stroke) in the YOD group than in the MD and SD groups.

Baseline clinical parameters, including baseline HbA1c, FBG and eGFR, were significantly greater in the YOD group than in the MD and SD groups, while systolic BP (SBP), low density lipoprotein (LDL-C), high density lipoprotein (HDL-C), total cholesterol (TC), serum creatinine (SCr) and serum uric acid (SUA) were significantly lower in the YOD group than in the MD and SD groups (P<0.05) (**Table 1**).

**Table 1:** Comparison of baseline clinical data among different disease-onset age groups.

Clinical data	YOD (*n*=718)	MD (*n*=5086)	SD (*n*=1768)	*χ^2^/H*	*P*
Age (years)	43.00 (39.00,50.00)	58.00 (53.00,61.00)	66.00 (64.00,68.00)	3607.078	<0.001
Sex				21.024	<0.001
Male,n (%)	329 (45.8)	1881 (37.0)	664 (37.6)		
Female,n (%)	389 (54.2)	3205 (63.0)	1104 (62.4)		
Smoking,n (%)	197 (27.4)	1408 (27.7)	571 (32.3)	14.284	0.001
Drinking,n (%)	145 (20.2)	913 (18.0)	314 (17.8)	2.336	0.311
High intensity activity				23.138	<0.001
No	568 (79.1)	4044 (79.5)	1496 (84.6)		
Yes	150 (20.9)	1042 (20.5)	272 (15.4)		
Sleep duration (hours per day)	8.00 (7.00,8.00)	7.00 (6.00,8.00)	7.00 (6.00,8.00)	41.050	<0.001
T2DM duration (months)	89.00 (38.75,162.25)	52.00 (22.00,99.00)	20.00 (8.00,39.00)	1148.347	<0.001
Family history,n (%)	220 (30.6)	1272 (25.0)	339 (19.2)	42.421	<0.001
Comorbidities,n (%)					
Hypertension	199 (27.7)	2408 (47.3)	946 (53.5)	137.491	<0.001
Coronary heart disease	38 (5.3)	387 (7.6)	172 (9.7)	15.451	<0.001
Kidney disease	41 (5.7)	203 (4.0)	38 (2.1)	21.141	<0.001
Stroke	30 (4.2)	488 (9.6)	256 (14.5)	65.681	<0.001
Complications,n (%)					
Retinopathy	159 (22.1)	992 (19.5)	250 (14.1)	32.015	<0.001
Diabetic foot	28 (3.9)	151 (3.0)	32 (1.8)	10.134	0.006
Neuropathy	43 (6.0)	261 (5.1)	46 (2.6)	22.407	<0.001
Diabetic nephropathy	283 (39.4)	1547 (30.4)	385 (21.8)	86.916	<0.001
Use of medications,n (%)					
Antihyperglycemic therapy	631 (87.9)	4437 (87.2)	1302 (73.6)	190.01	<0.001
Oral hypoglycemic drugs	469 (65.3)	3605 (70.9)	961 (54.4)	161.309	<0.001
Insulin	185 (25.8)	615 (12.1)	73 (4.1)	239.177	<0.001
Glucose monitoring	97 (13.5)	678 (13.3)	160 (9.0)	23.203	<0.001
Antihypertensive drugs	173 (24.1)	2097 (41.2)	838 (47.4)	114.813	<0.001
Clinical parameter					
Systolic BP (mmHg)	133.50 (123,146)	142.00 (130,157)	148.00 (135,161)	247.788	<0.001
Diastolic BP (mmHg)	82.00 (76,90)	83.00 (76,90)	82.00 (76,90)	7.743	0.021
BMI (kg/m^2^)	25.47 (23.43,27.84)	25.76 (23.58,28.02)	25.90 (23.59,28.34)	5.550	0.062
Waist-hip ratio	0.90 (0.85,0.94)	0.90 (0.86,0.94)	0.90 (0.86,0.95)	4.993	0.082
Laboratory index					
LDL-C (mmol/L)	3.11 (2.51,3.79)	3.25 (2.65,3.92)	3.28 (2.63,3.91)	10.705	0.005
HDL-C (mmol/L)	1.33 (1.12,1.61)	1.37 (1.17,1.66)	1.42 (1.19,1.71)	27.542	<0.001
Total cholesterol (mmol/L)	5.04 (4.39,5.82)	5.19 (4.53,5.95)	5.24 (4.56,5.96)	13.110	0.001
Triglyceride (mmol/L)	1.55 (1.04,2.35)	1.62 (1.14,2.37)	1.6 (1.14,2.3)	4.930	0.085
CREA (μmol/L)	66.00 (54.00,79.00)	66.00 (56.00,79.00)	69.00 (60.00,82.00)	58.713	<0.001
UA (μmol/L)	273.50 (220.75,338.25)	283.00 (237.00,340.00)	301.00 (253.00,357.00)	82.878	<0.001
HbA1c (%)	8.10 (6.60,10.10)	7.40 (6.40,9.00)	6.70 (6.00,7.80)	281.434	<0.001
FPG (mmol/L)	9.69 (6.83,13.17)	8.11 (6.39,10.69)	6.82 (5.7,8.76)	333.980	<0.001
eGFR (mL/min/1.73m^2^)	100.63 (84.83,115.54)	89.96 (76.37,104.75)	82.59 (70.95,94.92)	331.703	<0.001

The data are presented as the mean (SD), median (interquartile range), or n (%) as appropriate. The Kruskal−Wallis H test was used for comparisons between groups, and the chi-square (X^2^) test was used for all categorical count data. Two-by-two comparisons were made using the X^2^ split method. BMI, body mass index; HbA1c, hemoglobin A1c; HDL-C, high-density lipoprotein cholesterol; LDL-C, low-density lipoprotein cholesterol; eGFR, estimated glomerular filtration rate, calculated using the Chronic Kidney Disease Epidemiology Collaboration (CKD-EPI) equation.

### Survival analysis among different age groups at onset

**Figure 2:**
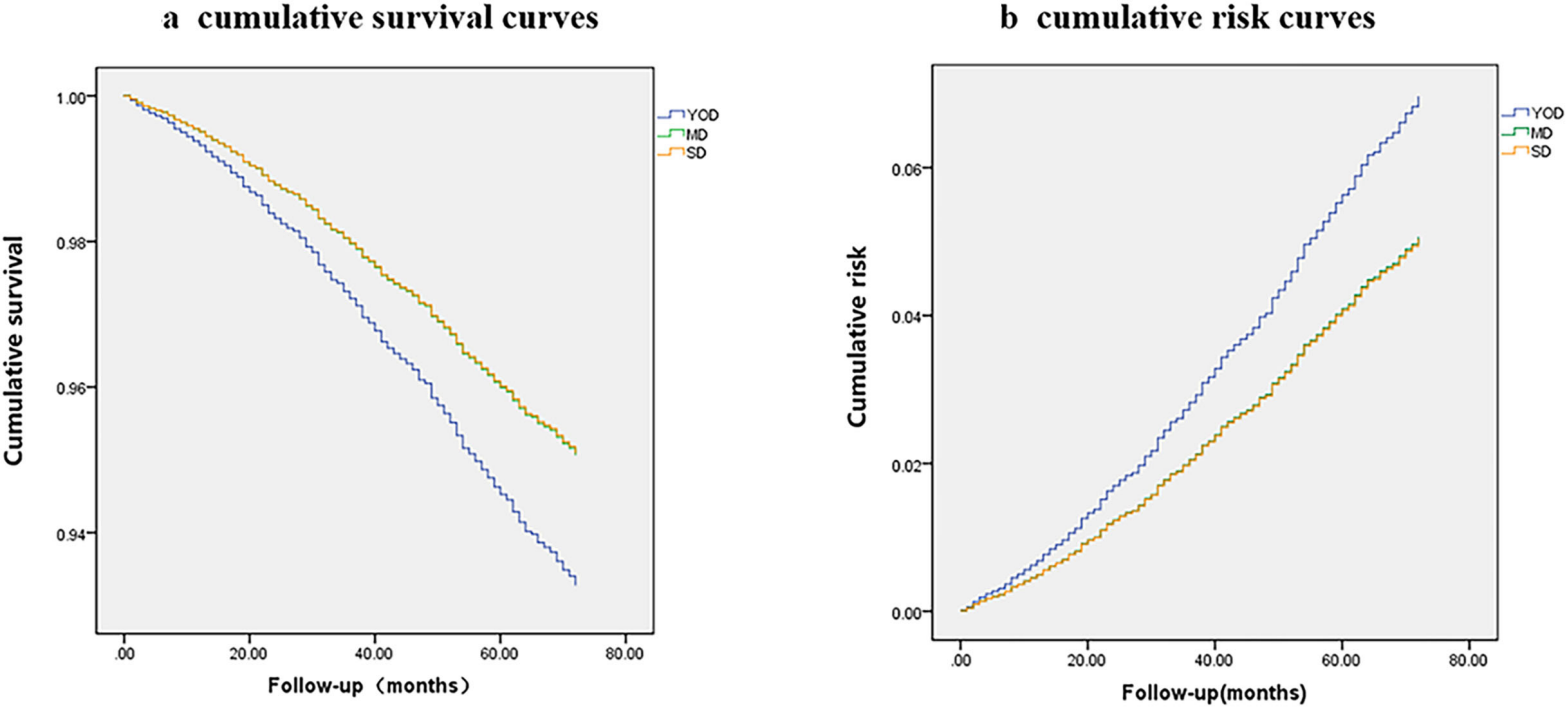
Cumulative survival curves **(A)** and risk curves **(B)** based on age at onset.

To explore the association between age at onset of T2DM and the risk of all-cause mortality in this study cohort, cumulative risk curves and survival curves were plotted (**Figure 2**), adjusting for age at onset, sex, high-intensity activity, comorbidities and complications, glucose-lowering treatment, and abnormal clinical parameters (SBP, LDL-C, HDL-C, TC, UA, HbA1c, and eGFR levels). The survival curve of the YOD group was lower than those of the MD and SD groups, which basically overlapped with each other (**Figure 2A**). However, the risk curve of the YOD group was higher than those of the MD and the SD groups, which also basically overlapped with each other (**Figure 2B**).

### Subgroup survival analysis among different age groups at onset

The present study also conducted survival analysis among different subgroups according to different age groups at disease onset.

The high risk of mortality in YOD patients was consistent in different subgroups according to sex, hypertension status (present or absent), coronary heart disease status (present or absent), antihypertensive treatment status (received or did not receive), glucose-lowering treatment status (received or did not receive), and alcohol consumption status (yes or no) (interaction P>0.05). However, the association between YOD and all-cause mortality was stronger in patients with a history of stroke and smoking (interaction P<0.05).

Among female patients, compared to those with SD, patients with YOD and MD had 97% (HR=1.978, 95% CI=0.777-5.333) and 17% (HR=1.179, 95% CI=0.800-1.738) greater risks of death, respectively. Among patients without a history of hypertension at baseline, compared to those with SD, patients with YOD and MD had a 65% (HR=1.659, 95% CI=0.679-4.053) and 19% (HR=1.191, 95% CI=0.792-1.791) increased risk of death, respectively. In patients without a history of coronary heart disease at baseline, compared to those with SD, patients with YOD had a 27% (HR=1.276, 95% CI=0.640-2.541) increased risk of death, while patients with MD had a similar risk of death (HR=0.991, 95% CI=0.740-1.327). Among patients who did not receive antihypertensive treatment at baseline, 40% (HR=1.406, 95% CI=0.596-3.319) and 4% (HR=1.047, 95% CI=0.715-1.540) greater risks of death were observed in the YOD and MD patients, respectively, than in the SD patients. Among patients who did not receive glucose-lowering treatment at baseline, 43% (HR=1.438, 95% CI 0.082-25.197) and 9% (HR=1.090, 95% CI 0.453-2.626) of the patients in the YOD and MD groups, respectively, had an increased risk of death compared with those in the SD group. Among T2DM patients without a history of alcohol consumption, patients in the YOD and MD groups had a 39% (HR=1.392, 95% CI 0.683-2.838) and 8% (HR=1.083, 95% CI 0.799-1.467) greater risk of death, respectively, than did the patients in the SD group (**Table 2**).

**Table 2:** Subgroup analysis of the association between age at onset of T2DM and all-cause mortality.

Subgroup	Diagnosis age group			P for interaction
	YOD	MD	SD	
Sex				0.990
Male	1.031 (0.410-2.597)	0.856 (0.576-1.271)	1	
Female	1.978 (0.777-5.033)	1.179 (0.800-1.738)	1	
Hypertension				0.601
Yes	1.196 (0.437-3.273)	0.885 (0.607-1.290)	1	
No	1.659 (0.679-4.053)	1.191 (0.792-1.791)	1	
Coronary heart disease				0.695
Yes	1.206 (0.120-12.177)	1.058 (0.460-2.435)	1	
No	1.276 (0.640-2.541)	0.991 (0.740-1.327)	1	
Stroke				<0.001
Yes	2.007 (0.392-10.290)	0.864 (0.471-1.584)	1	
No	1.266 (0.615-2.604)	1.044 (0.766-1.424)	1	
Antihypertensive therapy				0.573
Yes	1.282 (0.446-3.689)	0.975 (0.657-1.446)	1	
No	1.406 (0.596-3.319)	1.047 (0.715-1.540)	1	
Antihyperglycemic therapy				0.151
Yes	1.320 (0.670-2.603)	1.007 (0.752-1.349)	1	
No	1.438 (0.082-25.197)	1.090 (0.453-2.626)	1	
Drinking				0.072
Yes	1.279 (0.222-7.382)	0.745 (1.374-1.482)	1	
No	1.392 (0.683-2.838)	1.083 (0.799-1.467)	1	
Smoking				0.006
Yes	1.612 (0.481-5.402)	1.100 (0.690-1.754)	1	
No	1.316 (0.601-2.883)	0.991 (0.704-1.394)	1	

The data were adjusted for age, sex, education level, marital status, medical insurance status, smoking status, alcohol consumption status, high-intensity activity status, sleep duration, duration of T2DM, comorbidities (hypertension, coronary heart disease, kidney disease, stroke), BMI, antihypertensive treatment, glucose-lowering treatment, and laboratory parameters (eGFR, uric acid, glycated hemoglobin, high-density lipoprotein cholesterol). HR values are shown outside the parentheses, and 95% CI values are shown inside the parentheses.

### Sensitivity analysis

To address the issue of reverse causality arising from the presence of cardiovascular disease and other comorbidities at baseline, we performed sensitivity analyses by excluding patients with preexisting coronary artery disease, renal disease, and stroke at baseline, as well as individuals who died within the first 3 years of follow-up. The results showed that patients with YOD still had the highest risk of death (**Table 3**).

**Table 3:** Sensitivity analysis of the association between age at onset and all-cause mortality.

Group	Multivariable-adjusted hazard ratio (95% CI)			
	Excluding patients with coronary heart disease	Excluding patients with kidney disease	Excluding patients with stroke	Excluding patients who died within three years of follow-up
YOD	1.264 (0.632-2.527)	1.238 (0.617-2.482)	1.147 (0.557-2.359)	1.281 (0.660-2.665)
MD	0.973 (0.725-1.305)	1.010 (0.759-1.343)	1.010 (0.739-1.380)	0.991 (0.687-1.430)
SD	1	1	1	1

The data were adjusted for age, sex, education level, marital status, medical insurance status, smoking status, alcohol consumption status, high-intensity activity status, sleep duration, duration of T2DM, comorbidities (hypertension, coronary heart disease, kidney disease, stroke), BMI, antihypertensive treatment, glucose-lowering treatment, and laboratory parameters (eGFR, uric acid, glycated hemoglobin, high-density lipoprotein cholesterol). HR values are shown outside the parentheses, and 95% CI values are shown inside the parentheses.

### Analysis of risk factors for death in different age groups at onset

The shared risk factors contributing to all-cause mortality among the various age groups included age and stroke at baseline (**Table 4**, P<0.001). The risk of all-cause mortality increased by 7%-12% for each additional year of age at baseline. Conversely, BMI emerged as a protective factor (P<0.001).

**Table 4:** Analysis of risk factors affecting death in different age groups at disease onset.

Variable	YOD(n=718)			MD(n=5086)			SD(n=1768)		
	*HR(95% CI)*	*Waldχ^2^*	*P*	*HR(95% CI)*	*Waldχ^2^*	*P*	*HR(95% CI)*	*Waldχ^2^*	*P*
Age	1.07 (1.027-1.115)	10.362	0.001	1.072 (1.049-1.096)	39.230	<0.001	1.127 (1.054-1.205)	12.225	<0.001
Stroke	4.448 (1.677-11.801)	8.988	0.003	1.42 (1.065-1.895)	5.690	0.017	2.104 (1.496-2.959)	18.288	<0.001
BMI	0.847 (0.756-0.95)	8.071	0.004	0.918 (0.888-0.949)	25.799	<0.001	0.949 (0.909-0.991)	5.733	0.017
Uric acid	1.003 (1.001-1.006)	6.195	0.013	1.002 (1.001-1.003)	9.329	0.002	–	–	–
Diabetic foot	4.288 (1.731-10.62)	9.898	0.002	–	–	–	2.633 (1.277-5.427)	6.880	0.009
Sleep duration	1.305 (1.046-1.629)	5.545	0.019	–	–	–	–	–	–
Female	–	–	–	0.564 (0.435-0.731)	18.628	<0.001	0.514 (0.382-0.692)	19.317	<0.001
Insulin	–	–	–	1.962 (1.526-2.523)	27.565	<0.001	2.144 (1.285-3.576)	8.531	0.003
FPG	–	–	–	0.965 (0.94-0.992)	6.635	0.010	1.064 (1.029-1.099)	13.325	<0.001
Drinking	–	–	–	0.65 (0.458-0.923)	5.812	0.016	–	–	–
High intensity activity	–	–	–	0.636 (0.454-0.89)	6.945	0.008	–	–	–
Systolic BP	–	–	–	1.009 (1.004-1.014)	12.381	<0.001	–	–	–
eGFR	–	–	–	0.992 (0.987-0.998)	7.004	0.008	–	–	–
HbA1c	–	–	–	1.185 (1.129-1.244)	46.946	<0.001	–	–	–

Variables not listed in the table showed no significant impact on the risk of death (p>0.05) based on hazard ratio (HR) values and their 95% confidence intervals. BMI, body mass index; FPG, fasting plasma glucose; HbA1c, hemoglobin A1c; eGFR, estimated glomerular filtration rate, calculated using the Chronic Kidney Disease Epidemiology Collaboration (CKD-EPI) equation.

The independent risk factor for YOD was sleep duration; independent risk factors for MD included SBP and HbA1c. The independent protective factors were alcohol consumption, high-intensity exercise, and eGFR. FBG was an independent risk factor for SD (**Table 4**, P<0.001).

### Relationship between sleep duration and risk of all-cause mortality in different age groups at onset

**Figure 3:**
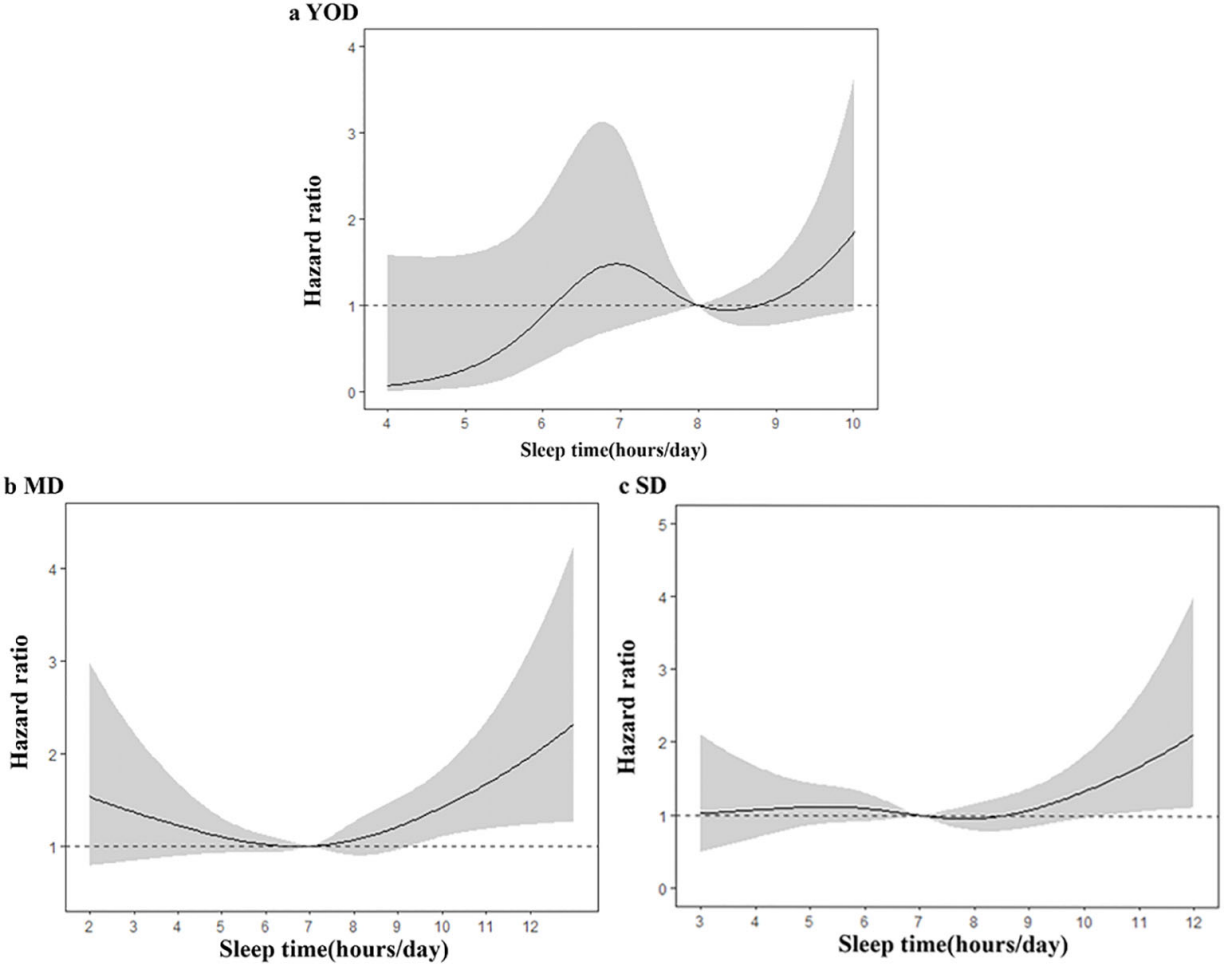
Dose−response Relationship between Sleep Duration and Risk of Death in T2DM Patients **(A–C)**. **(A)** YOD; **(B)** MD; **(C)** SD; adjustments were made for age, sex, education, marital status, health insurance status, smoking status, alcohol consumption status, high-intensity activity status, sleep duration, duration of T2DM, comorbidities (hypertension, coronary heart disease, kidney disease, stroke), BMI, antihypertensive therapy status, hypoglycemic therapy status, and laboratory indicators (eGFR, uric acid, HbA1c, high-density lipoprotein cholesterol).

Based on the above results, sleep duration was identified as an independent risk factor for high mortality risk in YOD patients. Restricted cubic spline analysis was used to examine the relationship between sleep duration and overall mortality risk in the three groups. The gray shading represents the 95% CIs associated with the HR values. For YOD, there was no clear nonlinear relationship between short sleep duration (less than 7 hours per day) and all-cause mortality. However, when the sleep duration exceeded 9 hours per day, the risk of death increased sharply. In the MD and SD groups, there was a J-shaped relationship between sleep duration and all-cause mortality. The lowest risk of death was observed at a sleep duration of 7 hours per day, and both shorter and longer sleep durations increased the risk of all-cause mortality in the MD and SD groups (**Figure 3**).

## Discussion

The present study analyzed the relationships between age at onset and lifestyle, complications, comorbidities, and clinical parameters in a cohort of patients with T2DM in China. The study also revealed the risk of mortality after a 6-year follow-up. A greater proportion of patients with YOD at baseline were engaged in high-intensity activities, had a longer sleep duration, had a longer duration of T2DM, had a greater proportion of patients with a family history of T2DM, had a greater proportion of microvascular complications (kidney disease, retinopathy, neuropathy, diabetic foot, etc.), and had a greater proportion of patients receiving glucose-lowering treatment. Moreover, patients in the YOD group also had significantly greater baseline HbA1c, FBG, and eGFR than did those in the MD and SD groups. After adjusting for relevant confounding factors, the baseline age in different age groups at onset was found to be an independent risk factor for mortality in T2DM patients. For each additional year of age, the risk of all-cause mortality increased by 7% to 12%. However, an early-onset age (i.e., the YOD group) significantly increased the risk of mortality in T2DM patients (HR=1.38, 95% CI=0.72-2.66), with a 1.38-fold greater risk in patients with YOD than in those with SD. Moreover, the risk of death in patients with YOD remained highest in the sensitivity analysis that excluded patients with coronary heart disease at baseline, stroke patients, and patients who died within the first three years of follow-up. In patients with YOD, sleep duration was identified as an independent risk factor for all-cause mortality, with a notable increase in the risk of all-cause mortality when sleep duration exceeded 9 hours per day.

There is limited research on the risk of mortality associated with YOD among Asian populations. The results of this study were similar to those of a cohort study conducted in Sweden with 318,083 T2DM patients (15). During a median follow-up of 5.63 years, patients diagnosed with T2DM at ≤40 years of age had the highest risk for most outcomes, with a more than doubled risk of all-cause mortality compared to that of the control group (HR=2.05, 95% CI=1.81-2.33). This risk was greater than that observed in our cohort, which may be due to the larger sample size in the Swedish study, which included 7,253 YOD patients. Hence, it is necessary to further expand the sample size to be able to observe more patients with YOD and their associated mortality risk in China.

Although YOD in China is associated with a higher risk of mortality than in Western countries, there are significant differences in many aspects. According to the subgroup analysis of this study, the risk of all-cause mortality in female YOD patients increased by 97% compared to that in the control group, while in male YOD patients, the risk increased by only 3%. This was contrary to the results obtained by researchers in Denmark (16). They reported that at any given age, the younger the age of diagnosis of T2DM is, the greater the risk of all-cause mortality in males, but the additional mortality risk is very weak in females. The possible reasons for this difference may be that the study cohort in Denmark was from the Steno Diabetes Center, which does not represent the general population of T2DM patients. In Denmark, most T2DM patients are treated by general practitioners, and only complex patients (with severe hyperglycemia or progressive diabetes complications) are referred to outpatient clinics. Hence, the results of the Danish study may be more applicable to complex T2DM patients treated in specialized centers.

In the subgroup analysis of this study, YOD patients with a history of hypertension had a 19% greater risk of all-cause mortality than did the control group, while YOD patients without a history of hypertension had a 65% greater risk. This finding was inconsistent with the results of a cohort study (17) from Singapore, which revealed that in T2DM patients younger than 65, the risk of cardiovascular disease mortality was significantly greater in the hypertension group than in the normal blood pressure group. The possible reasons for this discrepancy may be the difference in age grouping criteria between the two studies, or it could be because in this study, YOD patients with a history of hypertension had higher compliance with antihypertensive and glucose-lowering treatments, with proportions of 86% and 87%, respectively, compared to only 78% of T2DM patients receiving antihypertensive treatment in the Singapore cohort study. Since the proportion of patients receiving glucose-lowering treatment was not listed in the Singapore study, validation in different cohorts is needed in the future.

This study also revealed that regardless of baseline coronary heart disease status, YOD patients had a greater risk of all-cause mortality (interaction P value=0.695). In YOD patients without a history of coronary heart disease at baseline, the risk of all-cause mortality was greater than that in the control group (HR=1.276, 95% CI=0.640-2.541). This was in contrast to the results of a cross-sectional study of 957 patients with T2DM in northern Jordan (18), which showed that the risk of death in patients with T2DM who had coronary heart disease at baseline was 1.6 times greater than that of patients without coronary heart disease (OR=1.62, 95% CI=1.05-2.48). Potential explanations for this incongruity may stem from the limited sample size in the Jordanian study and the advanced age of the participants (mean age of patients was 60.99 ± 0.37). And cardiovascular disease has always been the leading cause of death in the middle-aged and the elderly with T2DM (19), but not in YOD. There is a paucity of research examining the relationship between the presence or absence of coronary heart disease at baseline and the risk of all-cause mortality in patients with T2DM. Therefore, future controlled studies with larger sample sizes are warranted. Moreover, regardless of whether baseline antihypertensive treatment was administered, YOD patients still had the highest risk of mortality. However, YOD patients receiving antihypertensive treatment had a slightly lower risk of all-cause mortality than those not receiving antihypertensive treatment. In patients with T2DM, antihypertensive treatment has been proven to be beneficial and can improve overall mortality rates in this population (20). This study also revealed that regardless of whether baseline glucose-lowering treatment was performed, YOD patients still had the highest risk of all-cause mortality. This finding is similar to the conclusion of a randomized controlled study conducted in Sweden (21), which suggested that good glycemic control may not be sufficient to reduce excess mortality rates in YOD patients.

This study also explored the risk factors for all-cause mortality. Common risk factors for mortality in different age groups were age and stroke at baseline. A randomized controlled trial involving 11,140 patients with T2DM showed that baseline age was independently associated with the risk of all-cause mortality, with a 56% increase in adjusted risk for every 5-year increase in age (22). This finding is similar to our study results, the risk of all-cause mortality increased by 7% to 12% for every 1-year increase in age, which was lower than that reported in other studies. This may be because that study included only patients aged 55 at baseline, which is slightly different from our inclusion criteria. Moreover, a common protective factor was BMI. A meta-analysis of 21 studies revealed a nonlinear correlation between BMI and all-cause mortality in patients with T2DM, with the lowest mortality risk observed in the overweight group at a BMI of 33 kg/m^2^ (23). In the present study, BMI showed a negative linear correlation with all-cause mortality, with a decreasing trend in mortality risk in all three groups as BMI increased. Potential explanations for this phenomenon included the substantial sample size utilized in the meta-analysis (n=407,270), the extensive geographic representation (USA, Europe, UK, Asia) and different races, while our analysis was conducted in Chinese patients with T2DM. Therefore, universal weight loss recommendations may not be conducive to reducing all-cause mortality in Chinese patients with T2DM. A systematic review showed that for every 1% increase in HbA1c in individuals with T2DM, the risk of all-cause mortality increases by 1.15 times (24). In the present study, HbA1c levels had no significant effect on the risk of death in patients with T2DM (P > 0.05), possibly because of the lack of individualized stratified analysis of HbA1c in different populations. The American Diabetes Association and American Heart Association recommend personalized HbA1c targets based on individual patient factors: <7% for most adults, <6.5% for young patients with long life expectancies and no significant heart disease, and less strict (<8%) for those with certain medical conditions (25). Different populations have different HbA1c targets, which have different impacts on all-cause mortality.

Finally, this study revealed a J-shaped relationship between sleep duration and mortality risk in the MD and SD groups; the lowest mortality risk was observed at 7 hours per day, and the risk of death increased regardless of whether the sleep duration was more or less than 7 hours per day. This finding was similar to the results of a large cohort study in China (26). However, we also observed that sleep duration was an independent risk factor for all-cause mortality in YOD patients. There is limited research on the relationship between sleep duration and mortality risk in YOD patients. A prospective cohort study conducted in the United States (27) revealed that extreme sleep durations (less than 7 hours or more than 7 hours per day) increased the risk of all-cause and cardiovascular disease mortality in patients with T2DM, especially those diagnosed at a younger age. In our research, after adjusting for confounding factors, there was a statistically significant association between sleep duration and YOD and mortality risk (P<0.05). The risk of death sharply increased when YOD patients slept more than 9 hours per day, but insufficient sleep did not increase the risk of death in YOD patients. The association between sleep duration and mortality risk was weakened in the MD and SD groups, which may be related to factors such as age, socioeconomic status, lifestyle habits, medication use, and comorbidities.

There were some limitations in the study. First, this study was conducted in a single center, and further research is needed in different regions of China. Second, the number of YOD patients in this cohort was only 9.4% of the total study population (n=718), which is relatively small. Third, this study only recorded data on lifestyle factors and baseline clinical parameters at baseline, without considering their potential changes during follow-up.

## Conclusions

In conclusion, baseline age is an independent risk factor for mortality in patients with T2DM, and the risk of all-cause mortality increases by 7% to 12% for each additional year of age. The risk of all-cause mortality in YOD patients was 1.38 times greater than that in MD and SD patients, and the risk of death increased with increasing sleep duration, especially when sleep duration exceeded 9 hours per day.

Our findings highlight the crucial role of age at onset in predicting T2DM outcomes. Clinicians must carefully factor in age when developing treatment and follow-up plans. Younger patients face greater health challenges and higher mortality risk, necessitating improved monitoring and personalized management. The study concluded that, reduced sleep duration did not significantly elevate the risk of all-cause mortality in YOD. However, sleep durations exceeding 9 hours were associated with a significant increase in this risk. Future research should incorporate diverse ethnic populations to validate and broaden the applicability of these findings. Our findings contest the prevalent belief that sufficient sleep universally benefits health. Currently, there is an absence of comprehensive guidelines for the lifestyle management of patients with early-onset T2DM. It is anticipated that this study will inform the formulation of future guidelines aimed at optimizing treatment strategies for these patients, thereby reducing mortality risk and enhancing their quality of life.

## Data Availability

The datasets used and/or analyzed during the current study are available from the corresponding author upon reasonable request. Requests to access these datasets should be directed to WH, huwen787878@163.com.
